# Adult H3K27M-mutant diffuse midline glioma with gliomatosis cerebri growth pattern: Case report and review of the literature

**DOI:** 10.1016/j.ijscr.2020.02.046

**Published:** 2020-02-28

**Authors:** Anudeep Yekula, Mihir Gupta, Nicholas Coley, Hoi Sang U

**Affiliations:** a Department of Neurosurgery, Massachusetts General Hospital Harvard Medical School, 185 Cambridge Street, Richard B. Simches Building 3rd Floor, Boston, MA, 02114, United States; b Departments of Neurosurgery, UCSD Health, 9300 Campus Point Drive #MC7893, La Jolla, CA, 92037-1300, United States; c Departments of Neuropathology, University of California, UCSD Health, 9300 Campus Point Drive #MC7893, San Diego, La Jolla, CA, 92037-1300, United States

**Keywords:** CT, computed tomography, CSF, cerebrospinal fluid, MRI, magnetic resonance imaging, WHO, World Health Organization, Diffuse midline glioma, H3K27M mutation, Gliomatosis cerebri, Brain tumor, Neurosurgical biopsy, Adult

## Abstract

**Introduction:**

H3K27M-mutant diffuse midline glioma is a recently classified unique entity predominantly affecting pediatric patients and rarely adults. The clinicopathologic features in adults remain poorly characterized.

**Presentation of case:**

A 36-year-old man presented with subacute progressive cognitive and visual deterioration, and hydrocephalus requiring ventricular shunting. MRI revealed a diffusely infiltrating lesion with a gliomatosis cerebri growth pattern, multiple foci of contrast enhancement, and diffuse leptomeningeal involvement. Suboccipital craniotomy with exploration of the posterior fossa revealed a subtle capsular lesion infiltrating into the choroid plexus. Although histologically low-grade, the tumor was found to have an H3K27 M mutation establishing the diagnosis.

**Discussion:**

In spite of diverse clinicopathologic characteristics, H3K27M-mutant diffuse midline gliomas are incurable, WHO grade IV lesions with poor prognosis. We discuss our case in the context of a review of published reports of H3K27-mutant diffuse midline gliomas in adults. Findings late in the disease course may mimic inflammatory or infectious pathologies radiographically, and low-grade infiltrative neoplasms histologically.

**Conclusion:**

The diverse clinical, radiographic and molecular features of H3K27M-mutant diffuse midline gliomas in adults remain to be completely characterized. A high index of suspicion is required to avoid missing the diagnosis. Early biopsy and detailed molecular characterization are critical for accurate diagnosis and patient counseling.

## Highlights

• H3K27M-mutant diffuse midline gliomas are incurable, WHO grade IV tumors.
• These gliomas predominantly present in children, and rarely in adults.
• The diverse features of these gliomas in adults remain incompletely characterized.
• Early biopsy and detailed molecular characterization are critical.

## 1 Introduction

H3K27M-mutant diffuse midline gliomas have a characteristic lysine (K) to methionine (M) substitution in either the H3F3A or HIST1H3B/C genes. Many of these cases were previously classified as brainstem gliomas or diffuse intrinsic pontine gliomas (DIPG) [[1], [2], [3], [4]]. These lesions may occur in a variety of midline locations including the thalamus, brainstem and spinal cord. In spite of having a broad spectrum of histopathological appearances, they correspond to WHO grade IV due to an aggressive, incurable course with poor prognosis [[5], [6], [7], [8], [9]]. The revised 2016 World Health Organization (WHO) classification of tumors of the central nervous system identified H3K27M-mutant diffuse midline glioma as a unique entity with distinct clinical behavior and molecular features. These H3K27 M mutant diffuse midline gliomas have been extensively described in children [6,[10], [11], [12]], but are rarely reported in adults [6,[13], [14], [15], [16], [17], [18], [19], [20], [21], [22], [23], [24], [25], [26]]. We present a rare case of an adult with an H3K27M-mutant diffuse midline glioma. The clinical, radiographic, histopathologic and molecular findings are then discussed in the context of a comprehensive review of the literature of adult H3K27M-mutant diffuse midline gliomas. This work has been reported in line with the SCARE [27] criteria.

## 2 Presentation of case

A 36-year-old male presented with two months of nausea, vomiting, headache, bilateral visual loss, hiccups, fatigue, and weight loss. He was found to have communicating hydrocephalus and papilledema requiring ventriculoperitoneal shunting. His visual symptoms recurred one month later, requiring shunt revision. Cerebrospinal fluid (CSF) chemistries and infectious studies were unremarkable. After experiencing one week of improvement, his vision again relapsed.

**Fig. 1:**
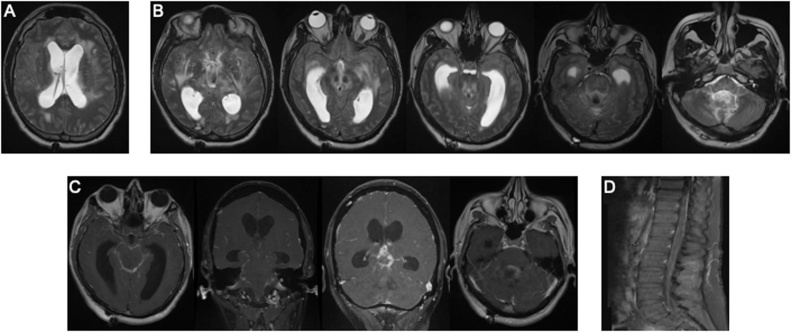
Magnetic resonance imaging of the neuraxis. A. Axial T2-weighted image showing hydrocephalus and transependymal flow. B. Representative T2-weighted axial images showing hyperintensity in bilateral insula, frontal lobes, posterior limbs of the internal capsules and thalami, extending inferiorly into the midbrain, pons, cerebellar peduncles, and medial cerebellar hemispheres. C. T1-weighted, contrast-enhanced images showing diffuse leptomeningeal enhancement and thickening within the basilar cisterns and upper cervical canal, pineal region and fourth ventricle. D. T1-weighted, contrast-enhanced sagittal scan of the thoracolumbar region showing diffuse leptomeningeal enhancement in the thecal sac.

**Fig. 2:**
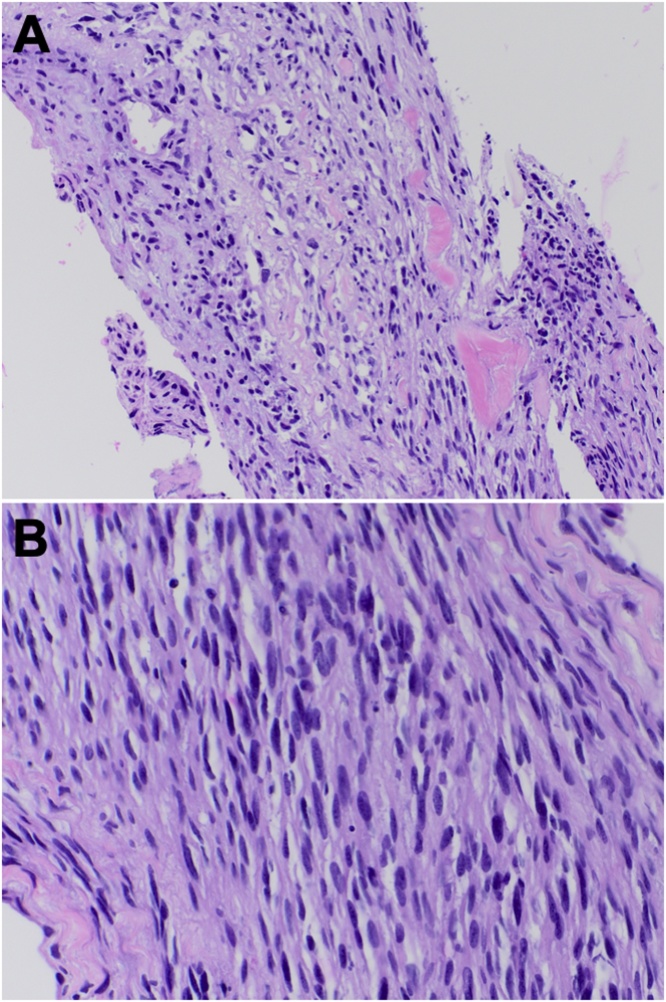
Hematoxylin and eosin (H&E) stain showing neoplastic spindle cell proliferation at 200× (A) and 400× (B) magnification.

On evaluation, the patient was drowsy, disoriented and blind in both eyes, without other focal deficit. Serum chemistries, blood counts and coagulation parameters were within normal limits. Magnetic resonance imaging (MRI) of the brain and spine was notable for communicating hydrocephalus and transependymal flow of cerebrospinal fluid (Fig. 1A). There was diffuse T2 hyperintensity involving the bilateral insula, frontal lobes, posterior limbs of the internal capsules and thalami, extending inferiorly into the midbrain, pons, cerebellar peduncles, and medial cerebellar hemispheres (Fig. 1B). Contrast-enhanced studies showed diffuse leptomeningeal enhancement and thickening most conspicuous within the basilar cisterns and upper cervical canal, with focal masslike leptomeningeal enhancement in the pineal region and within the fourth ventricle (Fig. 1C). Diffuse leptomeningeal enhancement was also observed within the thecal sac (Fig. 1D). MRA of the head and neck was grossly unremarkable (Fig. 2).

The differential diagnosis at this point included infectious etiologies such as meningitis and encephalitis; inflammatory conditions such as neurosarcoidosis and primary angiitis; and neoplastic etiologies such as diffuse astrocytoma with gliomatosis cerebri growth pattern and leptomeningeal spread. Contrast-enhanced computed tomography (CT) scans of the chest, abdomen and pelvis were unremarkable. Infectious and neoplastic assays on CSF were negative across multiple samplings. Another shunt revision was performed, with concurrent biopsy of the sural nerve and muscle. Biopsy results were negative, and the patient was thus offered craniotomy with exploration of the posterior fossa and open surgical biopsy.

A standard midline suboccipital craniotomy was performed. Upon opening the posterior fossa dura, thickened arachnoid was encountered and carefully divided. The cerebellar tonsils were split in order to expose the fourth ventricle. A small capsule in the wall of the fourth ventricle was biopsied; no other grossly abnormal area was encountered.

Histopathologic analysis of the fourth ventricular lesion showed neoplastic spindle cell proliferation with spread into the choroid plexus. Cells were positive for S-100 and GFAP, and rarely for CD68. Molecular genetic analysis showed an H3K27 M missense mutation in the H3F3A gene and a truncating mutation in the NF1 gene. Chromosomal analysis revealed multiple copy number losses (proximal 1p, 3p, 3q, and 10p; distal 10q, 11q, and 18p), one copy number gain (1p/q), monosomy 13, and one large region of loss of heterozygosity at 3q. An integrated diagnosis of H3K27M-mutant diffuse midline glioma was thus established.

Postoperatively the patient additional episodes of shunt failure requiring revisions. Due to continued clinical deterioration, his family elected to pursue palliative measures including whole brain radiation. He later suffered multiple episodes of aspiration pneumonia, and expired due to septic shock 8 months after initial symptom onset.

## 3 Discussion

The 2016 WHO classification of central nervous system tumors incorporated molecular parameters into the classification of diffuse gliomas, a dramatic change from the nearly century-old classification based exclusively upon histological features [28]. In the updated classification, H3K27-mutant diffuse midline gliomas comprise a distinct entity within IDH-wildtype gliomas, defined by a K27 M mutation in the H3F3A or HIST1H3B/C genes. In spite of sharing a common molecular perturbation, WHO grade IV designation and poor prognosis, these gliomas display tremendous phenotypic heterogeneity with respect to histologic, radiographic and clinical characteristics. Lesions predominantly affect the pediatric age group and have been infrequently reported in adults. The epidemiologic, radiographic, and clinical characteristics of H3K27M-mutant tumors in adults thus remain unclear. We describe a case of an adult H3K27-mutant diffuse midline glioma and present a comprehensive literature review of these gliomas in adult patients.

Published reports of adult H3K27-mutant diffuse midline gliomas are summarized in Table 1. The median age at diagnosis was 35 years (range 18–82 years). There was no gender preponderance. Clinical presentation correlated with anatomic location but was not reported in most studies. In children, H3K27M-mutant gliomas occurred frequently within the brainstem, particularly the pons, whereas in adults they were more frequently within the thalamus and spinal cord [21]. Rare adult cases were reported in the corpus callosum, hypothalamus, pineal region, basal ganglia and third ventricle. The diffuse, bilateral involvement of multiple deep structures with extension into the supra- and infratentorial white matter and leptomeninges as in our case was not previously reported, and may be due to the patient’s late presentation. Our report is thus a valuable vignette that may illuminate the natural history of this disease, implying progression to a gliomatosis cerebri growth pattern.

**Table 1:** Summary of the literature review of adult cases of H3K27M-mutant diffuse midline gliomas.

Author, Year	Meyronet, 2017	Solomon, 2016	Ebrahimi, 2019	Schreck, 2019	Wang, 2018	Kleinschmidt, 2018	Jiang, 2019	Enomoto, 2020	Aihara, 2013^a^	Yikui, 2019^a^	Daoud, 2018^a^	He, 2019
**Adult H3 K27 M gliomas, n**												
H3F3A K27M	20	NR	28	18	35	13	57	11	10	10	7	1
HIST1H3B K27M	1	NR	1	0	0	0	0	0	0	0	0	0
Total	21	20	29	18	35	13	57	11	10	10	7	1
**Age at diagnosis, years**												
Median	32	NR	37	38	NR	52	35	NR	38	32	41	
Range	18–82	NR	18-73	30-68	NR	2-81	NR	NR	17-46	18-54	25-54	
**Sex**												
Male	9/21	NR	19/29	8/18	19/35	4/13	34/57	NR	NR	6/10	6	
Female	12/21	NR	10/29	10/18	16/35	9/13	23/57	NR	NR	4/10	1	
**Clinical presentation**												
Intracranial hypertension	6/21	NR	NR	NR	NR	NR	NR	NR	NR	NR	NR	1
Ataxia	5/21											
Cranial Nerve Palsy	4/21											
Sensorimotor deficits	7/21											
**Location, n**^b^												
Midline cortex	0	NR	0	0	NR	0	0	NR				
Corpus Callosum	0		0	1/18	NR	0	0					
Spinal cord	6/21	NR	6/29	2/18	10/35	4/13	3/57	NR				
Thalamus	5/21	NR	15/29	3/18	7/35	7/13	33/57	NR	10	10		
Brainstem												
Midbrain	1/21	NR	NR	2/18	NR	0	NR	NR			2/7	
Pons	1/21	NR	NR	4/18	NR	1/13	NR	NR			2/7	
Floor of 4^th^ Ventricle	1/21	NR	NR	0	NR	0	NR	NR			2/7	
Medulla	2/21	NR	NR	0	NR	0	NR	NR			1/7	
Total brainstem	5/21	NR	7/29	6/18	11/35	1/13	15/57	NR			7	
Cerebellum	3/21	NR	1/29	6/18	NR	0		NR				
Hypothalamus	1/21	NR	1/29	0	NR	1/13	1/57	NR				1
Pineal region	1/21	NR	0	0	NR	0	5/57	NR				
Basal ganglia	0	NR	2/29	0	NR	0	0	NR				
3^rd^ Ventricle	0	NR	2/29	0	NR	0	0	NR				
Leptomeningeal involvement	1/18	NR	0/29	NR	NR	1/13	NR	NR	NR	NR	NR	0
Contrast Enhancement	9/18	NR	NR	15/18	NR	NR	46/57	NR	NR	NR	1/6	1
**WHO 2007 Grade**												
Grade I Grade II	03/21	NRNR	1/296/29	00	NRNR	1/131/13	NRNR	NRNR	00	NR NR	NR NR	
Grade III	0	NR	8/29	6/18	NR	7/13	NR	NR	4/10	NR	NR	
Grade IV	11/21	NR	14/29	11/18	NR	5/13	NR	NR	5/10	NR	NR	
Uncertain	7/21	NR	0	1/18	NR	0	NR	NR	0	NR	NR	
**Molecular Characteristics**												
IDH mutation	0	NR	0	0	0	NR	0	NR	0	0	0	0
MGMT promotor	1/21	NR	0	1/5	NR	NR	0	NR	1/7	∼17%	NR	NR
methylation	0	NR	NR	NR	NR	1/9	NR	NR	0	NR	NR	NR
EGFR amplification	0	NR	0	0	NR	NR	NR	NR	NR	NR	0	NR
BRAF V600E mutation	2/19	NR	NR	NR	NR	NR	1/27	NR	NR	0	NR	NR
TERT promotor	NR	NR	11/29	1/12	8/35	4/7	NR	NR	2/7	NR	0	1
mutation	NR	NR	0	NR	NR	NR	NR	NR	NR	NR	NR	0
ATRX mutation	11/21	NR	3/5	NR	26/35	NR	NR	NR	2/7	∼58%	1/6	1
ACVR1	21/21	NR	NR	NR	34/34	NR	NR	NR	NR	∼17%	NR	1
TP53	NR	NR	NR	NR	NR	3/7	NR	NR	NR	NR	NR	NR
Olig 2 expression	NR	NR	NR	NR	NR	NR	0	NR	NR	NR	NR	NR
PTEN Loss	NR	NR	NR	NR	NR	NR	NR	NR	3/7	NR	NR	NR
1p/19q Codeletion	NR	NR	NR	NR	NR	NR	NR	NR	1/7	NR	NR	NR
NF1	NR	NR	NR	NR	NR	NR	NR	NR	1/7	NR	NR	NR
CREBBP												
FGFR1												
**Extent of resection**												
GTR	0	NR	NR	1/18	NR	NR	15/57	NR	1/10	1/10	NR	1
STR	5/21	NR	NR	4/18	NR	NR	23/57	NR	1/10	5/10	NR	
PR/ Biopsy	16/21	NR	NR	12/18	NR	NR	18/57	NR	8/10	4/10	2/7	
Unknown	0	NR	NR	1/18	NR	NR	0	NR	0/10	0/10	2/7	
**Treatment**												
Surgery + RT + CT	12/21	NR	NR	11/18	NR	NR	28/57	NR	10/10	10/10	2/7	1
Surgery + RT	2/21	NR	NR	0	NR	NR	9/57	NR	0	0	0	
Surgery + CT	3/21	NR	NR	0	NR	NR	5/57	NR	0	0	0	
Surgery alone	3/21	NR	NR	0	NR	NR	15/57	NR	0	0	1/7	
RT alone	0	NR	NR	0	NR	NR	0	NR	0	0	0	
CT alone	4/21	NR	NR	0	NR	NR	0	NR	0	0	0	
CT + RT	0	NR	NR	0	NR	NR	0	NR	0	0	2/7	
Observation/Palliation	3/21	NR	NR	6/18	NR	NR	0	NR	0	0	0	
Unknown	0	NR	NR	1/18	NR	NR	0	NR	0	0	2/7	
**Post-operative complications**	NR	NR	NR	NR	NR	NR		NR	NR	NR	NR	
Cranial nerve palsy							21/57					
Motor deficit							11/57					
Sensory deficit							0					1
Ataxia							15/57					
**Median OS, months**	19.6	NR	16.1	17.6	NR	8.4	16	17	10.4	7	9	9

Abbreviations: ACVR1, activin A receptor type 1; ATRX, α thalassemia/mental retardation syndrome X-linked; CT, chemotherapy; CREBBP, CREB binding protein; EGFR, epidermal growth factor receptor; FGFR1, fibroblast growth factor receptor 1; GTR, gross total resection; IDH, isocitrate dehydrogenase; MGMT, O(6)-Methylguanine-DNA methyltransferase; NF1, Neurofibromin 1; NR, not reported; Olig 2, Oligodendrocyte transcription factor 2; PR, partial resection; OS, overall survival; PTEN, phosphatase and tensin homolog; RT, radiotherapy; STR, subtotal resection; TERT, telomerase reverse transcriptase; TP53, tumor protein 53.

a These series described cases exclusively arising in the brainstem (Daoud) or thalamus (Aihara, Yikui).

b Totals may exceed 100% because some lesions involve more than one anatomic location.

Reported radiographic appearances were also highly heterogeneous, though infrequently described in detail. Greater than 50% of cases displayed contrast enhancement on imaging. Leptomeningeal enhancement similar to our report was described in only two cases [11,21,23]. Macroscopic features were infrequently described, though in general H3K27M-mutant tumors share characteristics of other infiltrative gliomas including distortion and enlargement of affected structures, with some areas of necrosis, hemorrhage or discoloration. It remains unknown whether there are optimal radiographic or intraoperative features to target when contemplating surgical biopsy. Our report of careful posterior fossa exploration and attention to subtle abnormalities may thus serve to guide future operative planning. Furthermore, it remains imperative to have intraoperative pathologic confirmation of tumor-infiltrated tissue before concluding the procedure.

H3K27M-mutant gliomas display a wide spectrum of histologic phenotypes, and are classified as WHO grade IV irrespective of histologic grade [[5], [6], [7],^21^,^28^]. The lack of high-grade features occasionally suggests a low-grade astrocytic lesion, similar to our case. Importantly, all reported H3K27M-mutant gliomas in adults behaved aggressively irrespective of histologic grade, consistent with the WHO classification and reinforcing the need for molecular genetic analysis [6,17]. Indeed, existing reports of adult lesions were frequently limited by insufficient tissue and/or capacity for next-generation sequencing analysis of a wide panel of genes.

Under the WHO classification, H3K27M-mutant gliomas are by definition IDH-wildtype and lack 1p/19q co-deletion. These gliomas are instead characterized by a distinct mutation in histone H3 genes [4]. Mutations may occur in either the H3F3A or HIST1H3B/C gene; the former is more common in all age groups, but far more so in adult patients for unknown reasons, comprising 95–100% of existing reports [1,2,14,29,30]. Driver mutations remain unknown, and the exact mechanism of gliomagenesis due to these histone modifications is still unclear. Although the mutational burden is low, H3K27 M histone mutations have distinct and widespread effects on the epigenetic landscape, ultimately reprogramming the expression of myriad cancer-related genes [29,31,32]. Similar methylation patterns were identified in both adults and children, though differences in gene expression patterns are still being elucidated [13].

The majority of cases are managed with surgery, radiation and alkylating chemotherapy (primarily Temozolomide). The role of surgery is primarily for diagnosis, owing to the surgically inaccessible location of most lesions. Prognosis remains dismal even with timely and optimal treatment; overall survival is typically 7–11 months in children [11,22,33] and 8–19 months in adults [6,[13], [14], [15], [16], [17], [18], [19], [20], [21], [22], [23], [24], [25]], though published series to date are insufficiently powered to determine whether these differences are statistically significant [16,22,30]. It also remains unclear whether H3K27 M mutations independently affect prognosis compared to H3K27-wildtype status [[5], [6], [7],^13^,^14^,^22^,^34^]. The dramatic progression of the disease prior to our patient’s presentation highlights the need for early detection and initiation of treatment. Our aggregation of prior reports in adult patients will also serve as a benchmark to inform interdisciplinary treatment discussions with patients and families.

## 4 Conclusion

H3K27M-mutant diffuse midline gliomas predominantly affect pediatric patients, but may rarely present in adults. Our comprehensive literature review of adult cases highlights the need for further studies to characterize the diverse clinical, radiographic and molecular features of these lesions in adults. These gliomas must remain in the differential diagnosis of any new brain lesion affecting midline structures. Presentation late in the disease course may mimic gliomatosis cerebri growth pattern radiographically, and benign infiltrative neoplasms histologically. Early surgical biopsy with attention to subtle anatomic abnormalities, intraoperative histopathologic analysis, and molecular genetic analysis including histone mutation screening are critical for accurate diagnosis, management and patient counseling.

## Funding

This research did not receive any specific grant from funding agencies in the public, commercial, or not-for-profit sectors.

## Ethical approval

This is a case report; therefore, it did not require ethical approval from ethics committee.

## Consent

Exhaustive attempts were made to contact the family of the deceased patient. The paper has been sufficiently anonymized not to cause harm to the patient or the family. We have uploaded a separate document attesting to this, signed by the head of our medical team.

## Author contribution

All authors: writing the paper, design and data collection, data analysis and interpretation.

## Registration of research studies

Not applicable.

## Guarantor

Hoi Sang U, MD; and Mihir Gupta, MD.

## Provenance and peer review

Not commissioned, externally peer-reviewed.

## Declaration of Competing Interest

The authors report no conflicts of interest.
